# Development of a Dynamically Re-Configurable Radio-Frequency Interference Detection System for L-Band Microwave Radiometers

**DOI:** 10.3390/s24134034

**Published:** 2024-06-21

**Authors:** Adrian Perez-Portero, Jorge Querol, Andreu Mas-Vinolas, Adria Amezaga, Roger Jove-Casulleras, Adriano Camps

**Affiliations:** 1 CommSensLab-UPC, Universitat Politècnica de Catalunya-BarcelonaTech, and IEEC/CTE-UPC, 08034 Barcelona, Spain; 2 SigCom Group, Interdisciplinary Centre for Security, Reliability and Trust (SnT), University of Luxembourg, 29 Avenue JFK, L-1855 Luxembourg, Luxembourg; 3 Microwave Sensors and Electronics S.L., 08720 Vilafranca del Penedès, Spain; 4 Electrical and Communication Engineering Department, College of Engineering, UAE University, Al-Ain P.O. Box 15551, United Arab Emirates

**Keywords:** RFI, earth observation, radiometry, remote sensing, FPGA

## Abstract

Real-Time RFI Detection and Flagging (RT-RDF) for microwave radiometers is a versatile new FPGA algorithm designed to detect and flag Radio-Frequency Interference (RFI) in microwave radiometers. This block utilizes computationally-efficient techniques to identify and analyze RF signals, allowing the system to take appropriate measures to mitigate interference and maintain reliable performance. With L-Band microwave radiometry as the main application, this RFI detection algorithm focuses on the Kurtogram and Spectrogram to detect non-Gaussian behavior. To gain further modularity, an FFT-based filter bank is used to divide the receiver’s bandwidth into several sub-bands within the band of interest of the instrument, depending on the application. Multiple blanking strategies can then be applied in each band using the provided detection flags. The algorithm can be re-configured in the field, for example with dynamic integration times to support operation in different environments, or configurable thresholds to account for variable RFI environments. A validation and testing campaign has been performed on multiple scenarios with the ARIEL commercial microwave radiometer, and the results confirm the excellent performance of the system.

## 1. Introduction

Microwave radiometers measure microwave thermal radiation emitted by all bodies at a temperature above 0 K to measure the various physical properties of objects and environments [1]. It has applications in several fields, including remote sensing and meteorology. Satellite-based radiometry at L-band (1–2 GHz) is commonly used for soil moisture and water salinity measurements, especially within the frequency range of 1400 to 1427 MHz (or equivalently, wavelengths of approximately 21 to 21.4 cm). Due to the weak signals involved in microwave radiometry remote sensing (i.e., in microwave radiometry the signal measured is the background noise), one of the most significant challenges is Radio-Frequency Interference (RFI) [2,3]. RFI refers to the unwanted electromagnetic signals originating from various sources that can interfere with the desired measurements. RFI signals are emitted illegally in bands reserved for passive observations (in-band RFI), or legally in adjacent bands, but a fraction of their power leaks into the radiometer bandwidth (near-band effect), or from harmonics of emissions at a much lower frequency band, or from inter-modulation products (out-of-band effect). The presence of RFI can significantly degrade the accuracy and reliability of L-band microwave radiometry measurements due to their high sensitivity requirements, in the order of 1 K or less (−124 dBm over a band of 27 MHz). RFI can easily obscure the desired signals, leading to errors in the data interpretation. The presence of RFI signals increases the total power in the band, which translates into a positive bias in the measured Antenna Temperature (AT), and ultimately an error in the geophysical variable to be observed. For example, soil moisture maps contaminated by RFI show soils to be dryer than they actually are [4], while sea surface salinity maps affected by (small) RFI show a lower salinity.

Due to the nature of microwave radiometry, the observation band is expected to be comprised of white Gaussian noise only, with contributions from different bodies within the antenna footprint at different Brightness Temperatures (BTs) ($T_{b}$). For this reason, normality tests such as kurtosis are often used to check for interference that does not typically have Gaussian statistics. Kurtosis is the best statistical-based RFI detection algorithm for almost all kinds of interfering signals [5], although it is known that it has a blind spot for sinusoidal and chirp interfering signals of the 50% duty cycle, which can be overcome using, e.g., the Anderson–Darling technique, as proposed in [6]. When combined with Fourier transform, it is usually called spectral kurtosis [7] because the statistical test is applied per frequency bin. It can be mathematically computed as the ratio between the fourth central moment of a random variable and the square of its variance (second central moment). However, in practice, as thermal noise is zero-mean, non-central moments are equivalent and used for the calculation of the kurtosis. Depending on the characteristics of the receiver, the detection of RFI can be approached from different angles (i.e., depending on the quantization [8,9] or computational requirements [10]). Data acquisition and processing for microwave radiometers is usually implemented using a combination of a Radio Frequency (RF) front-end, typically a Software-Defined Radio (SDR), and a Field-Programmable Gate Array (FPGA) board, which allows for a modular approach regarding the reception chain and the Digital Signal Processing (DSP) stages. FPGA and SDR platforms have gained popularity in DSP due to their flexibility, programmability, parallel processing capabilities, and real-time processing performance. They offer the ability to implement and modify algorithms easily, handle computationally intensive tasks, and process data in parallel, resulting in improved speed and throughput. The hardware–software co-design approach allows for optimal system performance, while rapid prototyping and development cycles facilitate quick iterations and testing. Additionally, these platforms offer cost advantages through their flexibility, scalability, and elimination of expensive custom hardware designs. Previous works [11,12] have studied the performance of RFI detection using complex signal kurtosis in Microwave Radiometer (MWR), both simulated and hardware-in-the-loop implementations. In this manuscript, a Hardware Design Language (HDL) algorithm that implements RFI detection and mitigation for single-polarization ground-based microwave radiometers will be presented, along with details of the FPGA-based implementation. Extensive field campaigns with the Airborne Radiometer In L-band (ARIEL) commercial microwave radiometer were performed, and the validation and testing strategies are explored to showcase its capabilities.

## 2. Algorithm Design

The design of an effective RFI detection and mitigation algorithm for microwave radiometers requires careful consideration of various factors, including signal characteristics, computational efficiency, and adaptability to dynamic RFI environments. In this section are outlined the key components and methodologies employed in the design of the algorithm.

### 2.1. Overview

**Figure 1:**
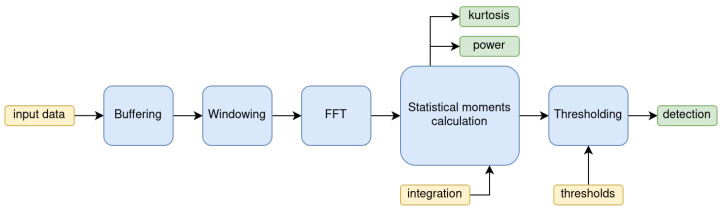
Block diagram of the RFI Detection HDL Block. In yellow, the user inputs (data, configuration), in blue, the RT-RDF stages, and in green the outputs.

The Real-Time RFI Detection and Flagging (RT-RDF) is an HDL block that can be implemented in either FPGAs or Application-Specific Integrated Circuits (ASICs). In this work, the target architecture is the Zynq Z7020 (Xilinx Inc., San José, CA, USA) System-on-Chip (SOC), with the Analog Devices AD9364 (Analog Devices, Norwood, MA, USA) SDR as the transceiver behind a custom frontend. Figure 1 presents a block diagram of the system architecture. Even though the HDL code itself is agnostic to the target architecture, the input has been tuned to work with 16-bit I/Q data from the 12-bit ADC. The range of possible values drives the inner fixed-point design, as well as the limits in possible integration times. The block has five distinct stages: buffering, windowing, Fast Fourier Transform (FFT), statistical moments calculation, and thresholding. The first three blocks prepare the signal to perform sub-band analysis using a filter bank, the statistical block computes the relevant tests for the detection of RFI, and the thresholding block provides the detection flags used for downstream applications. The following sections will provide greater detail about the different stages of the algorithm.

### 2.2. Detailed Algorithm Description

At the core of the proposed algorithm are state-of-the-art RFI detection and mitigation techniques, designed specifically for microwave radiometers. This algorithm leverages a combination of signal processing techniques (sub-band analysis) and statistical analysis (RFI detection) to identify and mitigate RFI signals while preserving the integrity of the radiometer’s measurements (blanking strategies).

#### 2.2.1. Sub-Band Analysis

The typical RFI found in L-Band MWR is narrowband [13]. This type of interference consists of narrow bandwidth (as compared in relation to the band of interest) and often appears as discrete spikes or lines on a spectrum. This can be caused by various sources, such as electronic devices, communication systems, or industrial equipment. Narrowband RFI typically has a bandwidth of a few kilohertz (kHz) to a few megahertz (MHz). For this reason, when detecting RFI and flagging data as corrupted, it is beneficial to do so in bandwidths that are a fraction of the 1400–1427 MHz frequency range. A typical solution to this segmented analysis of signals is known as a filter bank [7,9]. A filter bank refers to a set of filters that are used to separate and process signals within different frequency bands. It is an essential component of the signal processing chain for L-band radiometry. The purpose of a filter bank is to selectively filter out unwanted signals and interference while allowing the desired signals in the L-band frequency range to pass through. It helps to isolate the specific frequency components of interest and reduce the impact of RFI on the measured data. Even if the RFIs are wideband, this approach would help isolate the affected sub-bands if the detection algorithms in the following sections are able to flag them. The current RT-RDF implementation uses an FFT-based filter bank with 32 channels, which for the current receiver, spanning 24 MHz of bandwidth, results in 750 kHz sub-bands. When implementing the filter bank, it is necessary to use a 50% overlap window to reduce the spectral leakage, improve the frequency resolution, and reduce artifacts.

(1) $$w\left[k\right]=\sqrt{\frac{1}{2}\left[1-\frac{1-\beta }{\beta }\cdot \cos\left(\frac{2\pi k}{K}\right)\right]},\;\beta =\frac{24}{46},\;0\leq k\leq K$$

The window used in the implementation is a square-root Hamming window, defined as in Equation (1) [9]. To apply the window, the data must be read non-sequentially to obtain the overlap, compared to the arrival order. Thus, prior to the Windowing stage, the Buffering stage is used. After applying the normalized FFT in a serial configuration to reduce resource utilization, the 32 different channels are obtained.

#### 2.2.2. RFI Detection

The basic idea behind using statistical tools for RFI detection is that the statistical properties of RFI signals tend to deviate from those of the desired signals. RFI often exhibits non-Gaussian behavior, which means its statistical distribution differs from a Gaussian or normal distribution that is expected when measuring BT. Thus, methods that measure Gaussianity are very useful in detecting RFI for microwave radiometry. Kurtosis and [6], for instance, are computationally simple statistical measures used to describe the shape of a distribution, and they are often used in tests of Gaussianity to determine how much a given dataset deviates from a normal distribution. Kurtosis is a statistical measure that quantifies the peakedness of a probability distribution, whereas skewness is a measure of its asymmetry. Another common Gaussianity test is Anderson–Darling [6,14], which solves the blind spot of the kurtosis for sinusoidal and chirp RFI at 50% duty cycle [5], but its computational complexity is higher. Due to the real-time requirement and resource utilization constraints for the implementation of the algorithm, the chosen test is the kurtosis, as it is capable of detecting most common RFI [15], and as part of its calculation the power of the sub-bands can also be obtained and used together with the kurtosis as an indicator of the presence of interference. Power can be a powerful test against Gaussian RFI, as it can detect an abnormal increase in power, deviating from the expected values of the radiometer. This can be useful, for example, in detecting wideband RFI (e.g., communication links) that are Gaussian within their bandwidth.

Once the values of both kurtosis and power are obtained, a threshold needs to be applied to decide whether the signal is considered to be within the expected Gaussian and power bounds. The computation of the threshold depends on the distribution of the random variable that represents either the noise power or the kurtosis. The Neyman–Pearson Lemma [16] is a cornerstone of statistical hypothesis testing, providing a method for setting optimal thresholds in binary decision problems, such as signal detection in RFI scenarios. The lemma states that for a given significance level (the probability of a Type I error, or false alarm rate), the likelihood ratio test offers the most powerful test for distinguishing between two hypotheses: the null hypothesis ($H_{0}$), representing the absence of interference, and the alternative hypothesis ($H_{1}$), representing the presence of interference. By comparing the Probability Density Functions (PDFs) under the two hypothesis, the optimum threshold can be set. In practical applications, the performance of such tests can be visualized using Receiver Operating Characteristic (ROC) curves, which plot the true positive rate (sensitivity) against the false positive rate (1-specificity) for various threshold settings. The ROC curve provides a comprehensive view of a detector’s performance across different operating points. A key metric derived from the ROC curve is the Area Under the Curve (AUC). Maximizing the AUC is crucial because it quantifies the overall ability of the test to discriminate between the two hypotheses, regardless of the chosen threshold. An AUC of 1 indicates perfect discrimination, while an AUC of 0.5 suggests no discrimination, equivalent to random guessing.

By selecting a threshold based on this lemma, we can achieve the desired balance between the Probability of False Alarm (PFA) and missed detections, optimizing the performance of the detection system. Equation (2) shows an example of the theoretical computation of a threshold (α) for a certain number of samples, *N*, and a user-defined PFA [7] for the kurtosis of Gaussian noise. Once obtained, the threshold provides the maximum allowable excursion for the value from the expected mean (2 in the case of the kurtosis of a complex signal, 3 if real).

(2) $$\alpha_{\pm }=\frac{4}{N}\cdot \sqrt{2}\cdot erf^{-1}\left(1-2\cdot \mathrm{PFA}\right)$$

#### 2.2.3. Blanking Strategy

The RT-RDF algorithm provides the power and kurtosis values for the 32 sub-bands in the radiometer bandwidth. The user can then post-process the data and decide which blanking strategy to use. One of the possible strategies is to discard the corrupted sub-bands and average the valid ones. With this strategy, the radiometer would reduce its bandwidth dynamically as a function of the interference detected.

(3) $$\Delta T=\frac{T_{A}+T_{R}}{\sqrt{B\cdot \tau }}$$

Equation (3) states that the radiometric sensitivity (ΔT) of a total power radiometer is inversely proportional to the squared root of the bandwidth (*B*) times the integration time (τ); $T_{A}$ and $T_{R}$ being the antenna and the receiver’s noise temperature, respectively. For other microwave radiometer types, the formula will vary, but the term $1/\sqrt{B\cdot \tau }$ will still be present, and consequently the discussion and results hold for any type of microwave radiometer. Therefore, the maximum number of corrupted samples before discarding the entire sub-band needs to be a parameter decided by the radiometer’s user, who will adapt the maximum radiometric sensitivity to their specific application. In the case that the user needs the best radiometric resolution of the system [17], all the samples in the sub-band will be discarded, and thus the device will attempt to maintain the radiometric sensitivity with a lower number of sub-bands.

## 3. Validation and Results

The efficiency and performance of the proposed RFI detection and mitigation algorithm for microwave radiometers were evaluated through a series of comprehensive tests and validation procedures. In this section, we present the implementation, tests, and results of these experiments and validate the effectiveness of the algorithm in mitigating RFI interference while preserving the integrity of radiometric measurements.

### 3.1. Implementation Details

**Figure 2:**
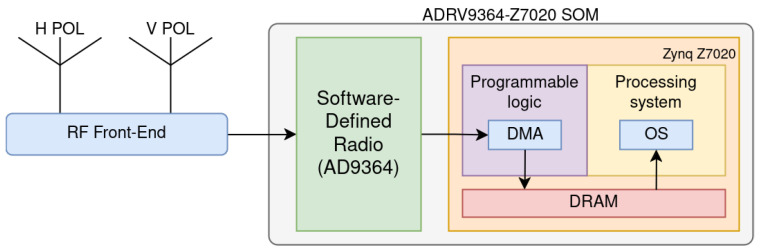
High-level block diagram of the radiometer, showing the antennas and RF frontend on the left and the processing stage within the FPGA on the right.

The RT-RDF algorithm was implemented in the ARIEL radiometer [18,19] to reduce the interference contributions coming from the environment and the aircraft themselves. The algorithm has been installed in the SOC as depicted in Figure 2. The resource utilization for the target architecture can be seen in Table 1. Because the algorithm has been implemented in an FPGA, it ensures consistent performance regardless of signal complexity. The FPGA’s architecture supports highly parallel processing, allowing for efficient management and processing of the input signal. Additionally, the deterministic design ensures that the system’s delay remains constant, providing predictable and stable performance, even under varying conditions. Thus, the performance remains constant, regardless of the amount of RFI present.

The instrument is an L-Band Total-Power Radiometer (TPR), which includes internal calibration loads (a hot load and a cold load) to compensate for intrinsic gain fluctuations and noise temperature drifts.

The data input of the algorithm is connected directly to the output of the Analog Devices AD9361 transceiver. The inputs of the user, such as the number of integration samples, kurtosis threshold, and power thresholds, are set by software using the registers shared between the FPGA and the processing unit. The outputs of the block are transferred to the memory using a Direct Memory Access (DMA). Once in the Random Access Memory (RAM), the data is processed and the chosen blanking strategy (see Section 2.2.3) is applied using the user’s input.

In the case of the ARIEL radiometer, the user can configure both the integration time of the radiometer and the RT-RDF using a Graphical User Interface (GUI). For the tests performed in this work, the number of integration samples is fixed at $2^{19}=524,288$ samples for the 32 sub-bands ($2^{14}$ samples per sub-band). This number ensures that the standard deviation of the hot load stays under 1 K (Equation (3)). It is assumed that the receiver’s noise temperature ($T_{R}$) is around 465 K and the antenna temperature ($T_{A}$) is around 290 K, as typical values. The power thresholds are not used in this implementation, so they are fixed to an arbitrary value. Regarding the kurtosis thresholds ($\alpha_{\pm }$), they have been decided using Equation (2), in which the PFA is related to the threshold through the number of integrated samples. The proposed PFA ($10^{-7}$) corresponds to a kurtosis threshold of 2±0.081 (complex kurtosis).

### 3.2. Test Campaign

In order to assess the effectiveness and performance of the RT-RDF, a test campaign was conducted. The primary objective of this campaign was to evaluate the algorithm under various real-world scenarios and RFI conditions, thus providing empirical evidence of its capabilities and limitations. The functionality of the radiometer with the RFI detector was tested in an interference-controlled environment, under three scenarios: one with no RFI to asses the impact in radiometric sensitivity, another with synthetic RFI with varying frequency, and a final scenario with synthetic RFI and varying power.

#### 3.2.1. RFI-Free Scenario

**Figure 3:**
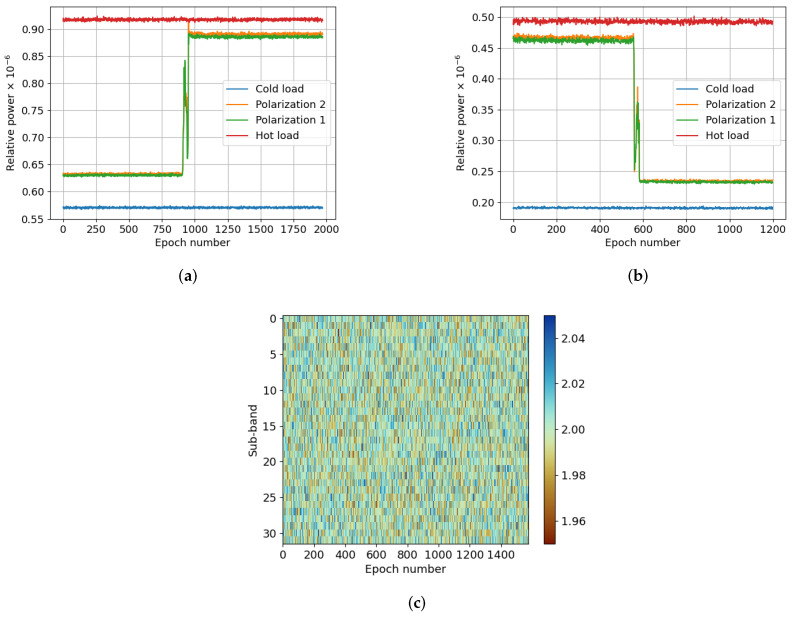
Data of the first measurements taken in an RFI-free environment. In (**a**), the power is captured without the RDDF algorithm enabled, and in (**b**) with it enabled. The antenna alternates between pointing at the microwave absorber (power closer to hot load) or the sky (power closer to cold load). (**c**) shows the kurtosis plot divided by sub-band and epoch number. (**a**) Radiometer’s output power, RFI mitigator OFF; (**b**) Radiometer’s output power, RFI mitigator ON; (**c**) Kurtosis.

The aim of the first test was to prove that the radiometric temperature detected when activating the RT-RDF is the same as when it is not used. Moreover, in both cases the linearity of the temperature as a function of the detected power shall be maintained. The value of this parameter is computed as the modulus squared of the samples coming out of the Analog to Digital Converter (ADC), and then calibrated using the internal loads. Additionally, the kurtosis statistics in an RFI-free scenario should be close to the theoretical values. That is, the mean complex kurtosis should be 2 and its standard deviation should be $\sigma_{kurt}\approx \sqrt{4/N}$, where *N* is the number of integrated samples in each sub-band. In these tests, $N=2^{14}$ and therefore $\sigma_{kurt}=0.01563$. Finally, the calibrated standard deviation of the hot load should be below 1 K. Figure 3a depicts the progression of the measured radiometric power without the RT-RDF. Initially, the antenna is pointed at the sky (cold values) before being re-positioned towards a microwave absorber (hot values). At L-Band, the sky’s BT is around 6 K, so the radiometer is more sensitive to RFIs. In Figure 3b, the RFI detector is enabled, and the antenna is now pointed first at the microwave absorber and then at the sky. Figure 3c presents the kurtosis value of the hot load measured during the acquisition.

Figure 3a,b show larger variability in the measured power with the RT-RDF enabled. These discrepancies can be attributed to differences in integration time between the cases with the block disabled and enabled (500,000 vs. 524,288, respectively), and to the presence of the filter bank. It can be seen that, in both cases, the linearity of the data in relation to the internal loads is preserved. The samples captured with the RT-RDF have a scaling factor that is corrected for the calibration to convert power to radiometric temperature, so the output is the same in both cases. The crucial metric to assess the quality of the data is the standard deviation of the radiometric temperature. Table 2 presents the standard deviations of the loads post-calibration, demonstrating highly consistent outcomes. It is important to note that the dynamic range of the MWR is preserved in both cases, as the difference between hot and cold loads is the same (0.563−0.363=0.2≈0.492−0.290). In Figure 3c, the progression of the hot load kurtosis across each sub-band is depicted. The computed mean value stands at 2.00007, with a standard deviation of $1.542\times 10^{-2}$, aligning well with theoretical expectations.

#### 3.2.2. Synthesized RFI, Frequency Sweep Test

**Figure 4:**
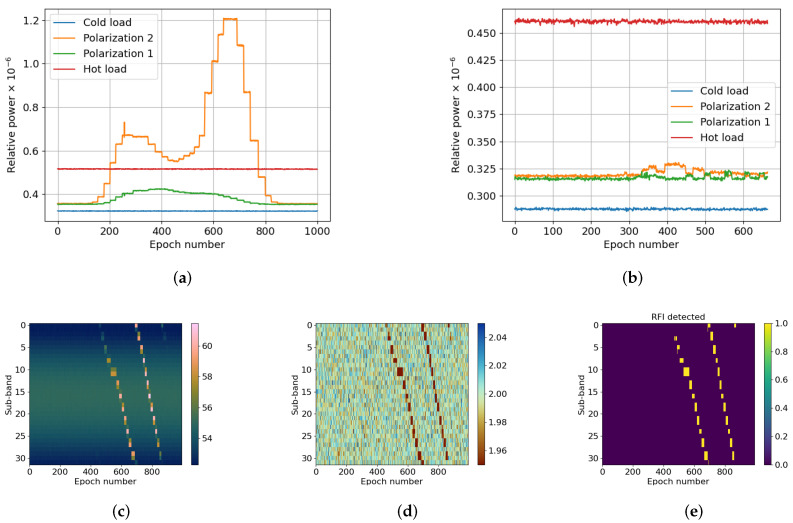
Data of the measurements taken in an RFI-free environment and sweeping the frequency of a narrowband interference. (**a**,**b**) depict the power detected by the radiometer when the RFI mitigator was disabled and enabled, respectively. (**c**–**e**) correspond to the power, kurtosis, and detection flag for the enabled case with respect to the epoch (i.e., duration of the test), respectively. (**a**) Radiometer’s output power, RFI mitigator OFF; (**b**) Radiometer’s output power, RFI mitigator ON; (**c**) Relative power (dB); (**d**) Kurtosis; (**e**) RFI detected.

**Figure 5:**
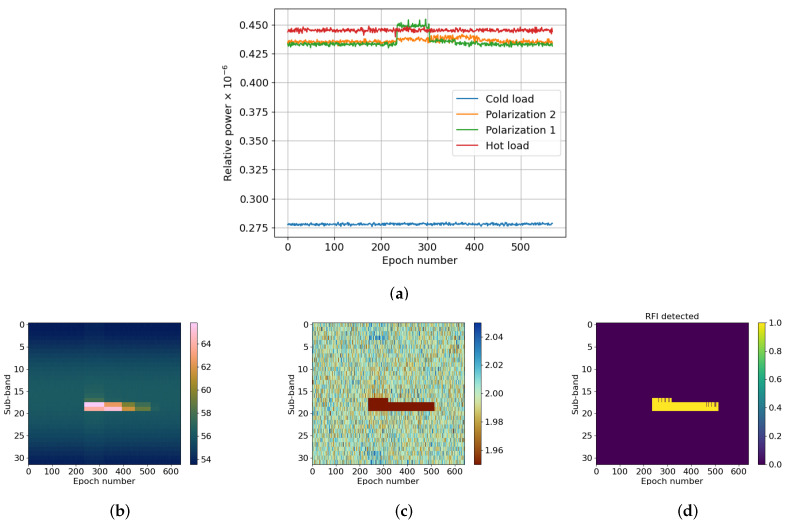
Data of the measurements taken in an RFI-free environment and decreasingly sweeping the power of a narrowband interference from −10 dBm to −80 dBm. (**a**) depicts the power detected by the radiometer when the RFI mitigator was enabled. (**b**–**d**) correspond to the power, kurtosis, and detection flag with respect to the epoch (i.e., duration of the test), respectively. (**a**) Radiometer’s output power; (**b**) Relative power (dB); (**c**) Kurtosis; (**d**) RFI detected.

Another test was performed to prove the ability of the system to detect and mitigate RFIs. The radiometer was pointed at the sky in the same RFI-controlled environment. Using a signal generator and an antenna, a single tone at 0 dBm was transmitted, sweeping the radiometer’s bandwidth. Figure 4a shows the power received by the radiometer during the test frequency sweep without the RT-RDF. Conversely, Figure 4b shows the same test with the mitigation block enabled. It can be clearly seen that most of the power of the interference is mitigated. Figure 4c,d,e depict the received power, kurtosis, and detection flag, respectively, for each sub-band. Despite successful mitigation of a significant portion of the interference power, some residual interference remains. Part of the power of the artificial interference is leaked to other sub-bands. The fact that this leakage does not have an impact on kurtosis makes the RT-RDF ineffective in detecting it. This phenomenon, although existent, cannot be seen in Figure 4b. However, between epoch numbers 220 and 320 in Figure 5b, the leakage in other sub-bands is clearly visible. Note that, in Figure 5d, the RT-RDF does not detect it. The test also revealed that the power of the two polarizations changed at different frequencies. The reason for this is the frequency response of the radiometer’s front-end, which varies the received power with the frequency. Another cause is the multipath effect, as it changes with frequency and the test was performed in an enclosed space without microwave absorber.

#### 3.2.3. Synthesized RFI, Power Sweep Test

The last test aimed to determine the maximum power level of an interference that the RT-RDF can mitigate without having an impact on the radiometric temperature. Using the same setup as in the previous tests, an interference was generated at a fixed frequency, sweeping its power from −10 dBm to −80 dBm. Figure 5a illustrates the power received by the radiometer after mitigating the RFI. Figure 5b depicts the power received in each sub-band, and Figure 5c the corresponding kurtosis measurement. Lastly, Figure 5d indicates the flagged sub-bands. The test results demonstrate that the power of the interference affects the capacity of the RFI detection block to mitigate it, performing better when the RFIs have lower power. Due to the large RFI power, the receiver is saturated and inter-modulation products appear at different bands, affecting the mitigation. When the power is lower, the system acts linearly and the RFI can be better detected with respect to the desired signal.

### 3.3. Results

Through extensive testing and analysis, we evaluated the effectiveness of the algorithm in distinguishing between RFI-affected signals and noise-only scenarios. The conducted frequency-sweep and power-sweep tests provided valuable insights into the performance of RFI detection algorithms under varying conditions. The frequency-sweep test involved systematically sweeping across different frequency bands, simulating the presence of RFI at different frequencies. The results from this test revealed the algorithm’s ability to detect interference across the different sub-bands.

Similarly, the power-sweep test evaluated the algorithm’s response to changes in signal power levels, mimicking scenarios where RFI exhibits different power characteristics. Analysis of the power-sweep test unveiled the algorithm’s robustness in detecting interference across a range of signal power levels, determining the maximum power level of an interference that the algorithm can mitigate without having an impact on the radiometric temperature.

As the detection and flagging is performed independently for each frequency band, complex environments containing multiple RFI with varying center frequencies and powers would not affect the capability of the algorithm to detect them with properly-configured thresholds. The results demonstrated the superiority of likelihood ratio-based thresholds in achieving a balance between false alarm rates and detection sensitivity.

## 4. Conclusions

The RT-RDF is an HDL algorithm designed to detect and mitigate RFI in microwave remote sensing instruments. It employs advanced algorithms and signal processing techniques for accurate interference detection and characterization. Configurability is a key aspect of the block, allowing instrument designers to customize thresholds, frequency ranges, and blanking techniques to optimize interference detection and minimize false alarms. The block’s integration is facilitated through comprehensive documentation covering resource utilization, interfaces, and integration considerations, ensuring smooth compatibility with other system components. The performance of the RT-RDF algorithm has been validated through testing, demonstrating reliable interference detection across various scenarios. Incorporating the block into electronic designs enhances system robustness and reliability, enabling effective mitigation of RFI and improved overall performance. However, mitigating RFIs effectively demands further investigation and the development of more sophisticated blanking algorithms to minimize the impact on measurements. The current implementation offers flexibility to end-users, allowing them to opt for using the block either as a detector or a mitigator. This decision hinges on the specific application and the desired level of radiometric sensitivity. Future enhancements may include adaptive interference mitigation, machine learning algorithms for automatic interference classification, and integration with system-level monitoring and control mechanisms. In summary, the RT-RDF algorithm provides a comprehensive, performant solution for detecting and mitigating RFI. Its configurability, accurate detection, and seamless integration capabilities make it a useful tool for instrument designers aiming to ensure reliable performance in the presence of interference.

## Figures and Tables

**Table 1:** Xilinx Vivado estimation of the utilized resources for a Zynq 7020 CLG400-1 FPGA.

Resource	Number	Available	Percentage
LUTs	4354	53,200	8%
Registers	5554	106,400	5%
BRAM Tiles	3	140	2%
DSPs	22	220	10%

**Table 2:** Standard deviation of the calibrated loads with and without the RFI Detection HDL Block.

Load	RFI Mitigator ON (K)	RFI Mitigator OFF (K)
Hot	0.563	0.492
Cold	0.363	0.290

## Data Availability

The datasets presented in this article are not readily available.

## Abbreviations

ADC Analog to Digital Converter; ARIEL Airborne Radiometer In L-band; ASIC Application-Specific Integrated Circuit; AT Antenna Temperature; AUC Area Under the Curve; BT Brightness Temperature; DMADirect Memory Access; DSP Digital Signal Processing; FFT Fast Fourier Transform; FPGA Field-Programmable Gate Array; GUI Graphical User Interface; HDL Hardware Design Language; MWR Microwave Radiometer; PDF Probability Density Function; PFA Probability of False Alarm; RAM Random Access Memory; RF Radio Frequency; RFI Radio-Frequency Interference; ROC Receiver Operating Characteristic; RT-RDF Real-Time RFI Detection and Flagging; SDR Software-Defined Radio; SOC System-on-Chip; TPR Total-Power Radiometer.
